# Supplementary material for: Multi‐dataset identification of innovative feature genes and molecular mechanisms in keratoconus
Source: J Cell Mol Med. 2024 Sep 19;28(18):e70079. doi: 10.1111/jcmm.70079 (PMC11412914; doi:10.1111/jcmm.70079)
Supplement: Supplementary file 2 — Material S2. [file JCMM-28-e70079-s002.zip › 7.GSEA analysis/1.ARL11/KEGG_PHOSPHATIDYLINOSITOL_SIGNALING_SYSTEM.html]

Details for gene set KEGG\_PHOSPHATIDYLINOSITOL\_SIGNALING\_SYSTEM[GSEA]

|  || Dataset | ARL11.group.cls#h\_versus\_l |
| Phenotype | group.cls#h\_versus\_l |
| Upregulated in class | h |
| GeneSet | KEGG\_PHOSPHATIDYLINOSITOL\_SIGNALING\_SYSTEM |
| Enrichment Score (ES) | 0.24036439 |
| Normalized Enrichment Score (NES) | 1.0398092 |
| Nominal p-value | 0.42137095 |
| FDR q-value | 1.0 |
| FWER p-Value | 0.998 |
Table: GSEA Results Summary

  

Fig 1: Enrichment plot: KEGG\_PHOSPHATIDYLINOSITOL\_SIGNALING\_SYSTEM      
 Profile of the Running ES Score & Positions of GeneSet Members on the Rank Ordered List

  

| SYMBOL | TITLE | RANK IN GENE LIST | RANK METRIC SCORE | RUNNING ES | CORE ENRICHMENT || 1 | PLCB2 | na | 134 | 0.619 | 0.0761 | Yes |
| 2 | PIK3R3 | na | 2180 | 0.284 | 0.0537 | Yes |
| 3 | INPP5J | na | 2685 | 0.256 | 0.0722 | Yes |
| 4 | PIK3CG | na | 2870 | 0.247 | 0.0988 | Yes |
| 5 | CALML6 | na | 3001 | 0.240 | 0.1261 | Yes |
| 6 | DGKE | na | 3504 | 0.219 | 0.1399 | Yes |
| 7 | PLCG1 | na | 4334 | 0.189 | 0.1404 | Yes |
| 8 | INPP4B | na | 4775 | 0.173 | 0.1500 | Yes |
| 9 | INPP5D | na | 5042 | 0.165 | 0.1636 | Yes |
| 10 | SYNJ1 | na | 5192 | 0.160 | 0.1800 | Yes |
| 11 | PIK3R1 | na | 6079 | 0.135 | 0.1719 | Yes |
| 12 | PLCG2 | na | 6096 | 0.135 | 0.1889 | Yes |
| 13 | DGKG | na | 6475 | 0.125 | 0.1941 | Yes |
| 14 | DGKD | na | 6734 | 0.118 | 0.2019 | Yes |
| 15 | DGKA | na | 7100 | 0.110 | 0.2055 | Yes |
| 16 | DGKQ | na | 7475 | 0.102 | 0.2079 | Yes |
| 17 | DGKZ | na | 8028 | 0.092 | 0.2039 | Yes |
| 18 | DGKB | na | 8228 | 0.088 | 0.2094 | Yes |
| 19 | PLCD4 | na | 8762 | 0.079 | 0.2042 | Yes |
| 20 | PIK3R2 | na | 8783 | 0.078 | 0.2137 | Yes |
| 21 | INPP5E | na | 9457 | 0.068 | 0.2031 | Yes |
| 22 | PIK3C2G | na | 9631 | 0.065 | 0.2065 | Yes |
| 23 | ITPR2 | na | 9655 | 0.065 | 0.2143 | Yes |
| 24 | PIP4K2B | na | 9697 | 0.064 | 0.2214 | Yes |
| 25 | PIK3R5 | na | 10118 | 0.059 | 0.2169 | Yes |
| 26 | ITPR1 | na | 10204 | 0.058 | 0.2219 | Yes |
| 27 | PLCB3 | na | 10228 | 0.057 | 0.2286 | Yes |
| 28 | ITPKB | na | 10340 | 0.056 | 0.2326 | Yes |
| 29 | DGKH | na | 10341 | 0.056 | 0.2398 | Yes |
| 30 | PRKCA | na | 10617 | 0.052 | 0.2386 | Yes |
| 31 | IPPK | na | 10781 | 0.050 | 0.2404 | Yes |
| 32 | PIKFYVE | na | 12989 | 0.027 | 0.1802 | No |
| 33 | PIK3CA | na | 13512 | 0.023 | 0.1681 | No |
| 34 | INPP5K | na | 13606 | 0.022 | 0.1682 | No |
| 35 | PTEN | na | 14202 | 0.017 | 0.1533 | No |
| 36 | CALM2 | na | 14606 | 0.014 | 0.1435 | No |
| 37 | PIP5K1C | na | 15509 | 0.007 | 0.1184 | No |
| 38 | DGKI | na | 15757 | 0.005 | 0.1120 | No |
| 39 | PIK3C2B | na | 16098 | 0.003 | 0.1026 | No |
| 40 | CALML5 | na | 17116 | -0.004 | 0.0737 | No |
| 41 | PRKCG | na | 17545 | -0.007 | 0.0623 | No |
| 42 | PLCD3 | na | 18060 | -0.011 | 0.0489 | No |
| 43 | ITPR3 | na | 18064 | -0.011 | 0.0501 | No |
| 44 | PLCB4 | na | 18133 | -0.011 | 0.0496 | No |
| 45 | CDS2 | na | 18145 | -0.011 | 0.0508 | No |
| 46 | INPPL1 | na | 18293 | -0.012 | 0.0481 | No |
| 47 | CALML3 | na | 18646 | -0.015 | 0.0399 | No |
| 48 | CALM1 | na | 18762 | -0.015 | 0.0385 | No |
| 49 | PLCD1 | na | 18962 | -0.017 | 0.0350 | No |
| 50 | CDIPT | na | 20760 | -0.029 | -0.0131 | No |
| 51 | PIK3C2A | na | 20771 | -0.030 | -0.0096 | No |
| 52 | ITPK1 | na | 20907 | -0.031 | -0.0095 | No |
| 53 | INPP1 | na | 21773 | -0.037 | -0.0297 | No |
| 54 | INPP5A | na | 22669 | -0.047 | -0.0494 | No |
| 55 | CALM3 | na | 22971 | -0.050 | -0.0517 | No |
| 56 | OCRL | na | 23094 | -0.051 | -0.0486 | No |
| 57 | PIK3C3 | na | 23449 | -0.055 | -0.0517 | No |
| 58 | PIK3CB | na | 23818 | -0.060 | -0.0546 | No |
| 59 | IMPA2 | na | 24172 | -0.065 | -0.0564 | No |
| 60 | IMPA1 | na | 24281 | -0.066 | -0.0510 | No |
| 61 | CDS1 | na | 24483 | -0.069 | -0.0479 | No |
| 62 | PI4KB | na | 25270 | -0.079 | -0.0603 | No |
| 63 | PIK3CD | na | 25759 | -0.087 | -0.0632 | No |
| 64 | PIP4K2C | na | 25761 | -0.087 | -0.0520 | No |
| 65 | PLCZ1 | na | 25918 | -0.089 | -0.0450 | No |
| 66 | PIP4K2A | na | 26510 | -0.099 | -0.0493 | No |
| 67 | PI4KA | na | 26536 | -0.099 | -0.0372 | No |
| 68 | INPP5B | na | 26907 | -0.106 | -0.0342 | No |
| 69 | ITPKA | na | 27014 | -0.108 | -0.0232 | No |
| 70 | SYNJ2 | na | 28733 | -0.150 | -0.0534 | No |
| 71 | INPP4A | na | 29575 | -0.174 | -0.0551 | No |
| 72 | PIP5K1A | na | 31287 | -0.231 | -0.0746 | No |
| 73 | PLCB1 | na | 32251 | -0.272 | -0.0672 | No |
| 74 | PIP5K1B | na | 33110 | -0.330 | -0.0494 | No |
| 75 | PLCE1 | na | 33309 | -0.344 | -0.0106 | No |
| 76 | PRKCB | na | 33863 | -0.399 | 0.0250 | No |
Table: GSEA details [plain text format]

  

Fig 2: KEGG\_PHOSPHATIDYLINOSITOL\_SIGNALING\_SYSTEM      
 Blue-Pink O' Gram in the Space of the Analyzed GeneSet

  

Fig 3: KEGG\_PHOSPHATIDYLINOSITOL\_SIGNALING\_SYSTEM: Random ES distribution      
 Gene set null distribution of ES for **KEGG\_PHOSPHATIDYLINOSITOL\_SIGNALING\_SYSTEM**
